# Haloalkaliphilic *Streptomyces* spp. AJ8 isolated from solar salt works and its’ pharmacological potential

**DOI:** 10.1186/s13568-015-0143-2

**Published:** 2015-08-27

**Authors:** John Selesteen Charles Adlin Jenifer, Mariathason Birdilla Selva Donio, Mariavincent Michaelbabu, Samuel Gnana Prakash Vincent, Thavasimuthu Citarasu

**Affiliations:** Centre for Marine Science and Technology, Manonmaniam Sundaranar University, Rajakkamangalam, Kanyakumari District, 629502 Tamilnadu India

**Keywords:** Antimicrobial, Haloalkaliphils, Non ribosomal peptide synthetase (NRPS), *Streptomyces* spp. AJ8

## Abstract

Antagonistic *Streptomyces* spp. AJ8 was isolated and identified from the Kovalam solar salt works in India. The antimicrobial NRPS cluster gene was characterized by PCR, sequencing and predict the secondary structure analysis. The secondary metabolites will be extracted from different organic solvent extraction and studied the antibacterial, antifungal, antiviral and anticancer activities. In vitro antagonistic activity results revealed that, *Streptomyces* spp. AJ8 was highly antagonistic against *Staphylococcus aureus, Aeromonas hydrophila* WPD1 and *Candida albicans.* The genomic level identification revealed that, the strain was confirmed as *Streptomyces* spp. AJ8 and submitted the NCBI database (KC603899). The NRPS gene was generated a single gene fragment of 781 bp length (KR491940) and the database analysis revealed that, the closely related to *Streptomyces* spp. SAUK6068 and *S. coeruleoprunus* NBRC15400. The secondary metabolites extracted with ethyl acetate was effectively inhibited the bacterial and fungal growth at the ranged between 7 and 19.2 mm of zone of inhibition. The antiviral activity results revealed that, the metabolite was significantly (P < 0.001) controlled the killer shrimp virus white spot syndrome virus at the level of 85 %. The metabolite also suppressed the L929 fibroblast cancer cells at 35.7 % viability in 1000 µg treatment.

## Introduction

Microbes from extreme environments have attracted considerable attention in recent years. This is primarily due to the secret that they hold about the molecular evolution of life and stability of the macromolecules. Majority of the studies on extremophilic organisms, however, have been confined to extremophilic bacteria and actinomycetes are relatively less explored group (Vasavada et al. 2006). Extreme environments are populated by groups of organisms that are specifically adapted to these particular conditions and these types of extreme micro-organisms are usually referred to as alkaliphiles, halophiles, thermophiles and acidophiles, reflecting the particular type of extreme environment which they inhabit (Horikoshi 1991). Study of extremophilic actinomycetes and identification of their metabolic properties are most important tasks in biotechnology (Vinothini et al. 2008).

Solar salterns are unique hypersaline environments, characterized by their high salt concentration and alkaline pH (Zafrilla et al. 2010). They are the important class of microbial resources and important producers of secondary metabolites including antimicrobials. Recent reports have revealed that salt requiring microbes are a robust source of new natural products and serve as model systems in drug discovery (Fencial 1997). Several actinomycetes, found to be proficient to produce antimicrobial compounds and halotolerant enzymes, have been reported from the coastal solar salterns (Vasavada et al. 2006; Aruljose et al. 2011). Actinomycetes are the most economically and biotechnologically valuable prokaryotes (Lam 2006) On the other hand, a great metabolic diversity and biotechnological potential has been found in halophilic and halotolerant microorganisms. *Streptomyces* are especially prolific and can produce a great many antibiotics (around 80 % of the total antibiotic production) and active secondary metabolites (Remya and Vijayakumar 2008).The past several decades many laboratories have conducted a vast screening effort to isolate secondary metabolites with interesting biological activities (Manivasagan et al. 2013). It is accepted that halophilic actinomycetes will provide a valuable resource for novel products of industrial interest, including antimicrobial, cytotoxic, neurotoxic, antimitotic, antiviral and antineoplastic activities (Cragg and Newman 2005).

In the realm of drug discovery, important new metabolites with biological activities have been and are still being discovered from actinomycetes and many of these are described as being produced by polyketide synthases (PKS) and NRPS. Nonribosomally synthesized peptides represent a large group of structurally complex metabolites that are manufactured from amino, hydroxy and carboxy acid monomers by large multifunctional enzymes, termed NRPS (Mootz and Marahiel 1997). They are modular mega-multifunctional enzymes that synthesize an incredibly diverse set of biological active peptides or cyclic lipopeptides (Schwarzer et al. 2003). They are antibiotics, biosurfactants, siderophores, and immunosuppressant, as well as antitumor and antiviral agents. These valuable biomolecules carry important medical and biotechnological applications (Roongsawang et al. 2005). The present study intends to the identification, NRPS characterization and the pharmacological influence of haloalkaliphilic *Streptomyces* spp AJ8 which isolated from solar salt works.

## Materials and methods

### Isolation of haloalkaliphilic *Streptomyces*

Mud sediments (depth 5 cm; salinity 260 ‰ & pH 10.5) was collected from the condenser pond of the solar salt works of Kovalam, Kanyakumari, Tamilnadu, India (8°05′04.35″N 77°31′17.07″E) during the summer season of May 2013. Ten grams of mud soil samples were suspended in 100 ml of sterile water and 0.1 ml of suspension from this was spread over 10 % NaCl concentration on Knight’s agar media (pH 7.2) and incubated at 28 °C for 2–3 weeks. The isolates was sub-cultured and maintained in slant culture at 4 °C as well as at 20 % (v/v) glycerol stock at −80 °C for future use.

### Preliminary in vitro antagonistic activity

Preliminary screenings of antagonistic activity were done by the method described by Shomurat et al. (1979) against pathogenic bacteria and fungi. The haloalkaliphilic actinomycetes strains were spot inoculated in starch casein agar medium for 4 days. After 4 days, then they were overlaid with 5 ml of sloppy agar (0.6 %) layer previously seeded with anyone of the test microbes, bacteria and fungi. Further this was incubated for 24 h at 37 °C and the diameter of the incubation zone was recorded in millimetres.

### Phenotypic identification and cultural characteristics

Based on the antagonistic activity, the best haloalkaliphilic actinomycetes strain was undergo the biochemical characterization following the International *Streptomyces* Project (ISP) procedures (Shimizu et al. 2000). The physiological characteristics of the isolates such as, growth at different pH (5.5, 6 0.5, 7.5, 8.5, 9.5 and 10.5), NaCl concentration (2, 4, 6, 8 and 10) were recorded in starch casein broth. A set of cultural characteristics was also examined using media and the ISP procedures recommended by Shirling and Gottlieb (1966). Mature aerial mycelium and substrate mycelium pigmentation were recorded on Starch casein agar media following incubation at 28 °C for 28 days.

### Genomic identification

One hundred nanogram of genomic DNA was isolated from *Streptomyces* spp. AJ8 and amplified by PCR using 16S rRNA universal primers. The PCR product was cloned into the vector pTZ57R and used to transform *Escherichia coli* DH5α. The transformants were sequenced using an ABI 3700 automated DNA sequencer. Sequences were compared with other 16S rRNAs obtained from GenBank using the BLAST program. The phylogenetic tree was constructed by Geneious 5.4.6 software and evolutionary history was inferred using the UPGMA method. The evolutionary distances were computed using the Maximum Composite Likelihood method (Arif et al. 2011) and are in the units of the number of base substitutions per site.

### PCR amplification of biosynthetic cluster gene NRPS

NRPS gene was amplified from the genomic DNA template of *Streptomyces* spp. AJ8 using the degenerate primer oligonucleotides sets namely A3: (5′GCSTACSYSATSTACACSTCSGG3′) and A7R: (5′SASGTCVCCSGTSCGGTAS3′). PCR reactions were performed in a final volume of 50 µl containing 10 % of extracted DNA, 0.4 µM of each primer, 0.2 mM of each of the four dNTPs, 5 µl extracted DNA, 1 U Taq polymerase with its recommended reaction buffer, and 10 % DMSO. PCR parameters were 95 °C for 5 min of initial denaturation, 95 °C for 30 s of denaturation, 59 °C for 2 min of annealing, 72 °C 4 min and 72 °C of 10 min for final elongation on Eppendorf Mastercycler personal, Germany. The PCR products were resolved in 1 % (w/v) agarose gels stained with ethidium bromide.

### Sequencing and database analysis

The PCR product was purified by using gel extraction kit (Medox Biotech India Pvt. Ltd.) and sequenced using an ABI 3700 automated DNA sequencer by M13^+^ and M13^−^ primers (Sambrook et al. 1989). Sequences were compared with other NRPS gene obtained from GenBank using the BLAST program. Nucleotide sequence alignment and identity was analysed by using Clustal X (1.8.1) software (European Bioinformatics Institute).The phylogenetic tree was constructed by Geneious 5.4.6 software and tree build by using the UPGMA method and the genetic distance calculated using Tamura-Nei method (Sneath and Sokal 1973).

### NRPS protein structure prediction analysis

In order to study the secondary structure and functional prediction, Iterative Threading Assembly Refinement (I-TASSER) online bioinformatics software was used. This algorithm modeled and worked based on LOMETS multiple-threading alignment and TASSER iterative simulation. I-TASSER server results predict accurate structure and function base on state-of-the-art algorithm. This server was ranked as No 1 server proved by current CASP7 and CASP8 experiment (Roy et al. 2010; Zhang 2008). After run the sequence in the protein 3D structure was downloaded and visualized using molecular visualization tools of RasMol. Quality of the predicted protein models was estimated using C Score and the calculation is based on Z-Score of threading alignment in LOMETS multiple-threading alignment and cluster density of I-TASSER simulation. TM and C Score were determined the structure similarity and confidence between the predicted protein model and the native protein structure (Barrett 1997). Ligand binding site also determined for binding the drug molecules to bind the cluster.

### Extraction of secondary metabolites

The selected haloalkaliphilic actinomycete, *Streptomyces* spp. AJ8 was inoculated into starch casein broth and incubated at 28 °C on a shaker (200–250 rpm) for 7 days. The culture broth was filtered through 0.45 μm membrane filter (Millipore Millex-HV Hydrophilic PVDF) and the filtrate was extracted with ethyl acetate, chloroform and methanol (1:1v/v) and shaken vigorously for 1 h in a solvent extraction funnel. Solvent and filtrate mixture were stabilized for 24–48 h and separated from aqueous phase. The extracts were concentrated in a rotary evaporator and lyophilized (Al-Hulu et al. 2012).

### Pharmacological influence of secondary metabolites

In vitro antibacterial and antifungal activity was performed by the secondary metabolites extracted from different solvents against pathogenic bacteria using agar diffusion following the method described by Holt et al. (1994). Antiviral activity was performed against White Spot Syndrome Virus (WSSV) following the method of Balasubramanian et al. (2006). The secondary metabolite incubated WSSV suspensions (29 °C for 3 h) were injected with intramuscularly to the Indian white shrimp, *Fenneropenaeus indicus.* Hemolymph was bled from the shrimps after the 3rd day of injection, and extracted the genomic DNA (Chang et al. 1999). Double step diagnostic PCR were performed from the genomic DNA template using the WSSV VP28 primer designed by Namita et al. (2007) and standard PCR protocols were followed (Takahashi et al. 1996). Anticancer activity was performed in L929 fibroblast cell lines treated with different concentrations (100, 500 and 1000 µg) of *Streptomyces* spp AJ8 yielded secondary metabolites and incubated for 24 h (Freshney 2006).

### Data analysis

One way Analysis of Variance (ANOVA) was carried out using SPSS statistics data package. Means were compared at 0.001 % level.

## Results

### In vitro antagonistic activity

Among the different *Streptomyces* spp isolated from the haloalkaliphilic origin, the *Streptomyces* spp. AJ8 had the potent antagonistic activity against various bacterial and fungal pathogens. The antagonistic activity recorded of 21.1, 14.4, 12.4, 11.1, 11.5 and 9.8 mm of zone of inhibition against the bacterial pathogens, *S. aureus, A. hydrophila* WPD1*, B. subtilis, E. coli* and *V. harveyi* respectively. They also suppressed the fungal sp ranged between 9 to 11.5 mm of zone of inhibition (Table 1).

**Table 1:** In vitro antagonistic activity of *Streptomyces* spp. AJ8 against bacterial and fungal pathogens

Sl. no	Bacterial/fungal pathogens	Zone of inhibition (mm)
1	*Escherichia coli*	11.1 ± 1.0
2	*Staphylococcus aureus*	21.1 ± 0.6
3	*Bacillus subtilus*	12.4 ± 0.4
4	*Aeromonas hydrophila* WPD1	14.4 ± 0.4
5	*Vibrio harveyi*	9.2 ± 0.4
6	*Aspergillus niger*	9.8 ± 0.3
7	*Candida albicans*	11.5 ± 0.2
8	*Pythium* spp.	8.1 ± 0.2

### Phenotypic identification and cultural characteristics

The morphological, biochemical and physiological confirmative tests revealed that, the *Streptomyces* spp. AJ8 strain was Gram positive, non motile, MR positive, VP negative, negative for indole production and H_2_S production etc. They also ferment glucose, sucrose, mannitol, starch and sorbitol etc. Due to the haloalkaliphilic nature, the strain was able to grow well up to 8 % NaCl and 10.5 pH etc (Table 2). Macroscopic observations revealed that, they mostly grow well in starch casein agar, glycerol aspargin agar, nutrient agar, Knight’s agar, yeast extract malt extract agar, actinomycetes agar and tyrosin agar etc. The aerial, substrate mycelium and pigmentations were found brownish colours in all growth media (Table 3).

**Table 2:** Biochemical and physiological characteristics of *Streptomyces* spp. AJ8 isolated from Kovalam solar salt works in comparison with reference *Streptomyces* strains

Sl. no	Confirmative tests	AJ8^a^	Reference strains		
			AJ9^a^	RJ1^b^	RJ2^c^
1	Grams staining	+	+	+	+
2	Motility	Non-motile	Non-motile	Motile	Motile
	Biochemical tests				
3	Methyl Red (MR)	+	+	+	–
4	Voges Proskauer (VP)	–	–	–	–
5	Indole production	–	–	–	–
6	Nitrate reduction	+	+	+	+
7	TSI test	+	+	+	+
8	H_2_S production	–	–	–	–
9	Urease activity	–	–	–	–
10	Starch hydrolysis	+	–	+	–
Carbon source (1 % w/v)					
11	Glucose	+	+	+	+
12	Sucrose	+	+	+	+
13	Maltose	+	+	+	+
14	Mannitol	+	+	+	+
15	Starch	+	+	+	–
16	Sorbitol	+	+	+	–
Nitrogen sources (1 % w/v)					
17	Histidine	++	+	+	+
18	Valine	++	+	++	+
19	Alanine	++	+	+	+
20	Methionine	+	+	++	+
21	Tryptophan	+	+	+	+
Effect of pH					
22	5.5	+	+	+	+
23	6.5	+	+	+	+
24	7.5	+	+	++	++
25	8.5	++	++	+	+
26	9.5	+++	+	–	–
27	10.5	+	–	–	–
Effect of NaCl concentration (w/v)					
28	2	++	+	++	++
29	4	++	+	++	++
30	6	+++	++	+++	+++
31	8	++++	+++	++	++
32	10	++	++	+	+

^a^ *Streptomyces* spp. AJ8

^b^ *Streptomyces* spp. AJ9: Jenifer et al. (2013); *Streptomyces* spp. RJ1

^c^ *Streptomyces* spp. RJ2: Remya (2013). All reference strains were isolated from solar salt works

**Table 3:** Cultural and morphological characteristics of *Streptomyces* spp. AJ8 on different media

Media	Culture characteristics			
	Growth	Aerial mycelium	Substrate mycelium	Pigmentation
Starch casein agar	Good	Ash	Dark brown	Brownish red
Glycerol Aspargin agar (ISP 5)	Moderate	Brown	Reddish brown	Brown
Nutrient agar	Good	Reddish brown	Light ash	Light brown
Knight’s agar	Abundant	Brown	Dark brown	Brownish red
Yeast extract malt extract agar (ISP2)	Fair	Brown	Light brown	Light brown
Actinomycetes isolation agar	Good	Brown	Reddish brown	Dark brown
Tyrosin agar (ISP 7)	Fair	Ash	Dark brown	Light brown

### Genomic identification

**Fig. 1:**
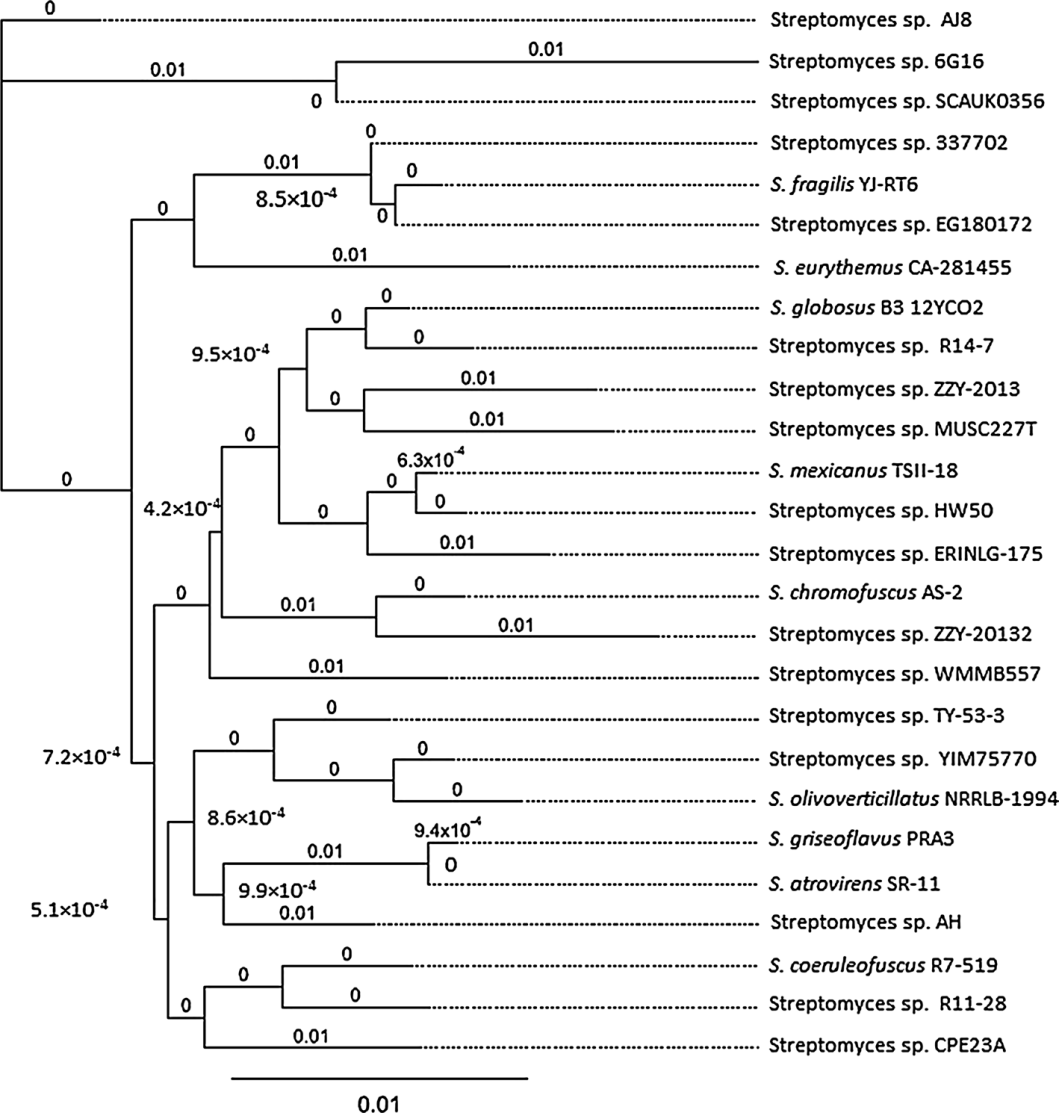
Graphical phylogenetic tree of halopalkaliphilic *Streptomyces* spp. AJ8 based on 16S rRNA gene sequence data compare with other *Streptomyces* spp. The tree was constructed using the HKY genetic distance model with neighbor-joining method

Phylogenetic and evolutionary analysis of the 16S rRNA sequence revealed that, *Streptomyces* spp. AJ8 shared high similarity to other *Streptomyces* spp strains including *Streptomyces* spp. 6G16, *Streptomyces* spp. SCAUKO356, *Streptomyces* spp. 337702, *S. fragilis* YJ-RT6 and *Streptomyces* spp. EG180172 (Fig. 1). The strain was deposited in NCBI database and the strain name and GenBank accession number are *Streptomyces* spp. AJ8, KC603899.1 respectively.

### PCR amplification and sequencing of NRPS gene

**Fig. 2:**
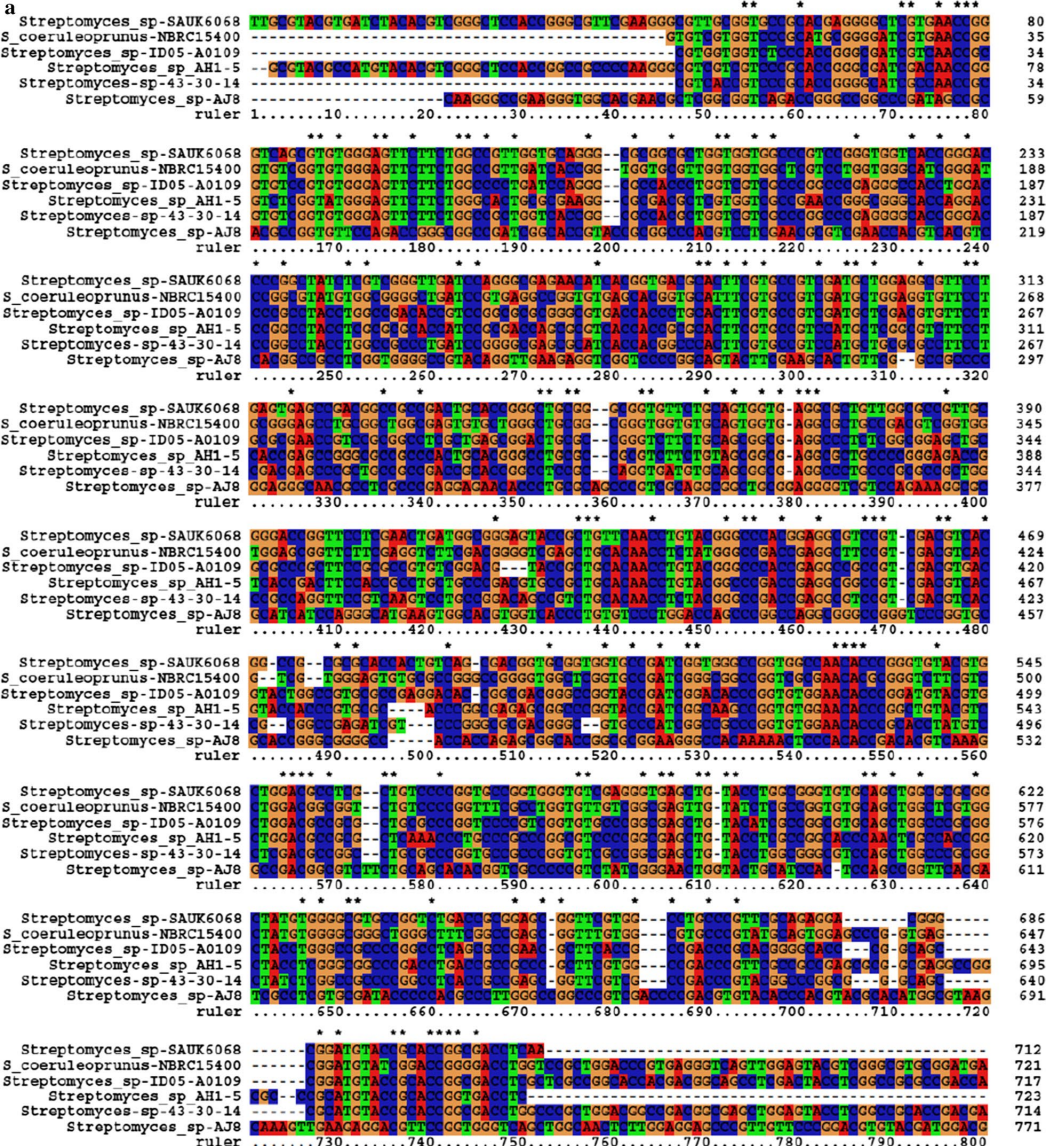
Nucleotide alignment identities of NRPS gene of haloalkaliphilic *Streptomyces* spp. AJ8 with its homologues. *Asterisks* indicate 100 % similarity among the nucleotide sequences

**Fig. 3:**
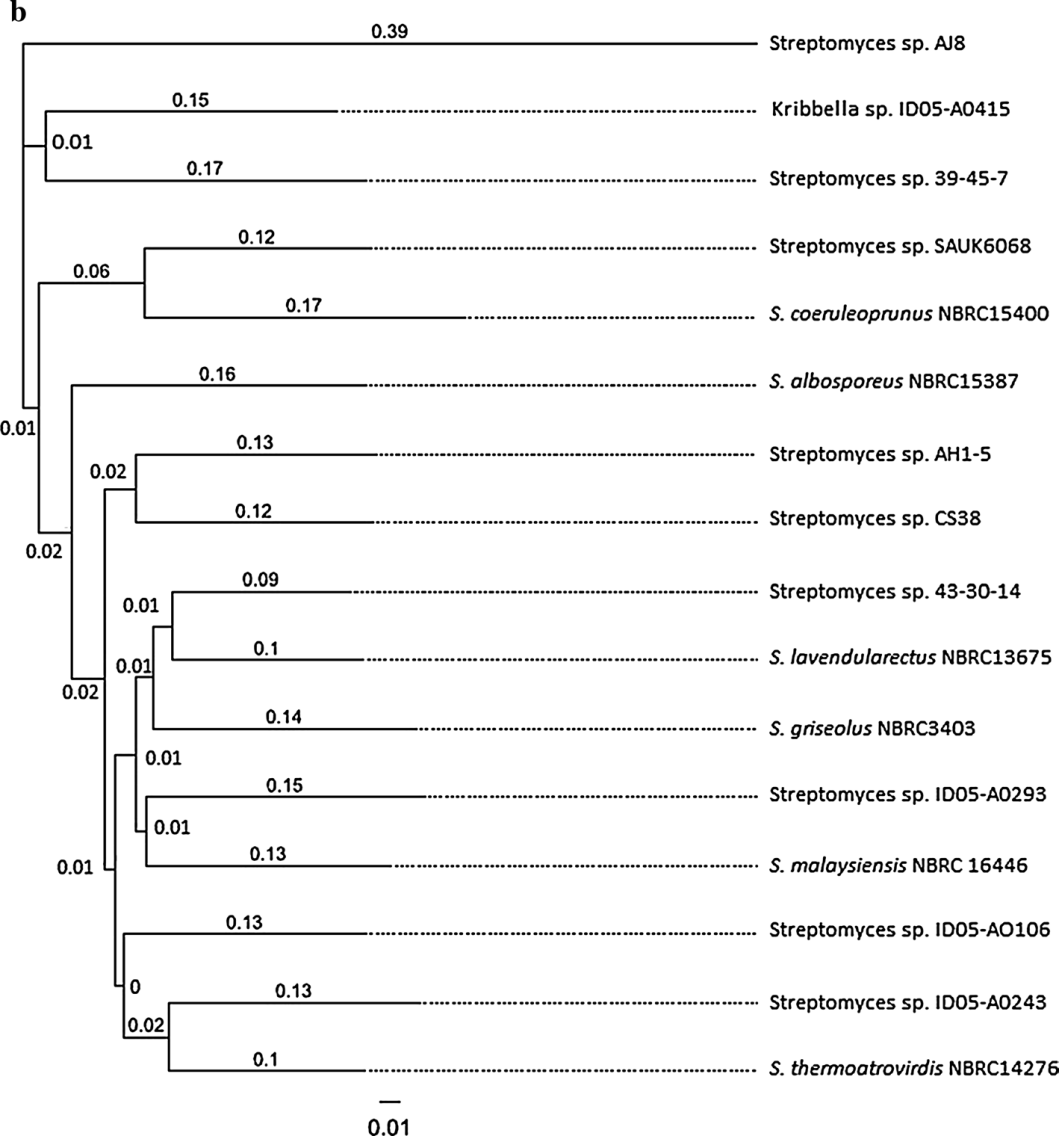
Phylogenetic analysis of the nucleotide sequence of NRPS gene of haloalkaliphilic *Streptomyces* spp. AJ8 with that reported in other *Streptomyces* spp. by Neighbor Joining method using Geneious Pro analysis

The NRPS gene specific PCR amplification generated a single gene fragment of 781 bp length and submitted to NCBI database (GenBank: KR491940.1). Multiple sequence alignment analysis revealed that, the NRPS gene of *Streptomyces* spp. AJ8 was more identical to the other NRPS family in *Streptomyces* spp. SAUK6068, *S. coeruleoprunus* NBRC15400, *Streptomyces* spp. AH1-5 and *Streptomyces* spp. 43-30-14 (Fig. 2). Phylogenetic and evolutionary analysis of the NRPS gene sequence revealed that, *Streptomyces* spp. AJ8 shared more than 80 % similarity to other *Streptomyces* spp. including *Streptomyces* spp. 35-45-7, *Streptomyces* spp. SAUK6068, *S. coeruleoprunus* NBRC15400, *Streptomyces* spp. NBRC15387 and Kribella spp. ID05-A0415 (Fig. 3).

### NRPS protein structure prediction analysis

**Fig. 4:**
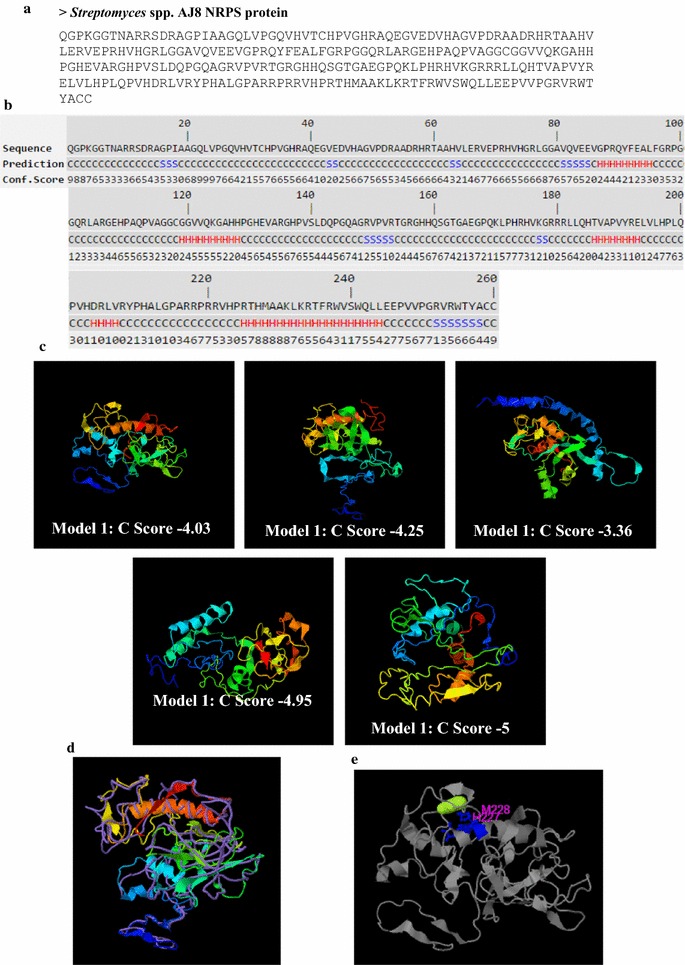
**a** FASTA format of NRPS protein query sequence. **b** Secondary Structure Prediction of the NRPS protein of *Streptomyces* spp AJ8. (*Color indications* *H* Helix; *S* Strands and *C* Coil). **c** The predicted 3D model and the estimated global and local accuracy of the NRPS protein. **d** The structure alignment of NRPS between the first I-TASSER model and the top 5 most similar structure templates in PDB. **e** The predicted ligand-binding sites of the NRPS protein. (Fluorescent *green yellow colour* indicated the predicted ligand binding site)

The Fig. 4a, b shows the protein sequence and the predicted secondary structure of NRPS by I-TASSER analysis. The top five models predicted territory structure for NRPS protein by I-TASSER and the C-score were −4.03, −4.25, −3.36, −4095 and −3.36 respectively (Fig. 4c). The estimated TM-score and RMSD observed of 0.28 ± 0.09 and 15.8 ± 3.2 Å respectively. The TM-align structural alignment results revealed that, the top five PDB hits were 4nl6A, 1qonA, 2c3mB, 2fj0A and 1n35A and its TM scores were 0.843, 0.442, 0.432, 0.426 and 0.426 respectively. The five top most aligned proteins among the NRPS were spliceosome, acetylcholinesterase, ferredoxin oxidoreductases (PFOR), esterase and reovirus polymerase etc. (Fig. 4d). The multiple binding site ligands from different PDB hits (1zeiF, 2xmbA, 4ekdA, 3bz1H and 2hi8X) were confirmed as M-Cresol, Beta-l-Fucose, Cobalt (2+), Chlorophyll *a* and Bromide etc. (Fig. 4e; Table 4). The predicted EC numbers of the PDB hits were given in the Table 5. Based on the results revealed that, the five top most PDB enzyme hits were dipeptidyl carboxypeptidase Dcp, mouse acetylcholinesterase, Cys-418 thiylradical, human acetylcholinesterase and recombinant human butyrylcholinesterase.

**Table 4:** Showing the ligand binding sites of the NRPS protein of *Streptomyces* spp AJ8

Rank	C-score	Cluster size	PDB hit	Ligand name	Downloaded complex	Ligand binding site residues
1	0.05	3	1zeiF	CRS	Rep, Mult	227, 228
2	0.05	3	2xmbA	FUL	Rep, Mult	200, 203, 204, 212, 215
3	0.03	2	4ekdA	CO	Rep, Mult	223, 227
4	0.02	1	3bz1H	CLA	Rep, Mult	236, 239
5	0.02	1	2hi8X	BR	N/A	86, 103, 104

**Table 5:** The predicted enzyme commission numbers and active sites *o*f the NRPS protein

Rank	C-score^EC^	PDB hit	TM-score	RMSD^EC^	IDEN^EC^	Cov	EC number	Active site residues
1	0.065	1y79A	0.419	6.15	0.074	0.719	3.4.15.5	NA
2	0.065	1maaD	0.404	6.29	0.048	0.704	3.1.1.7	NA
3	0.065	1h16A	0.416	6.33	0.042	0.727	2.3.1.54	NA
4	0.065	1b41A	0.345	6.35	0.030	0.623	3.1.1.7	NA
5	0.065	2pm8A	0.406	6.39	0.046	0.727	3.1.1.8	NA

*EC* Enzyme commission

### Pharmacological influence of secondary metabolites

**Fig. 5:**
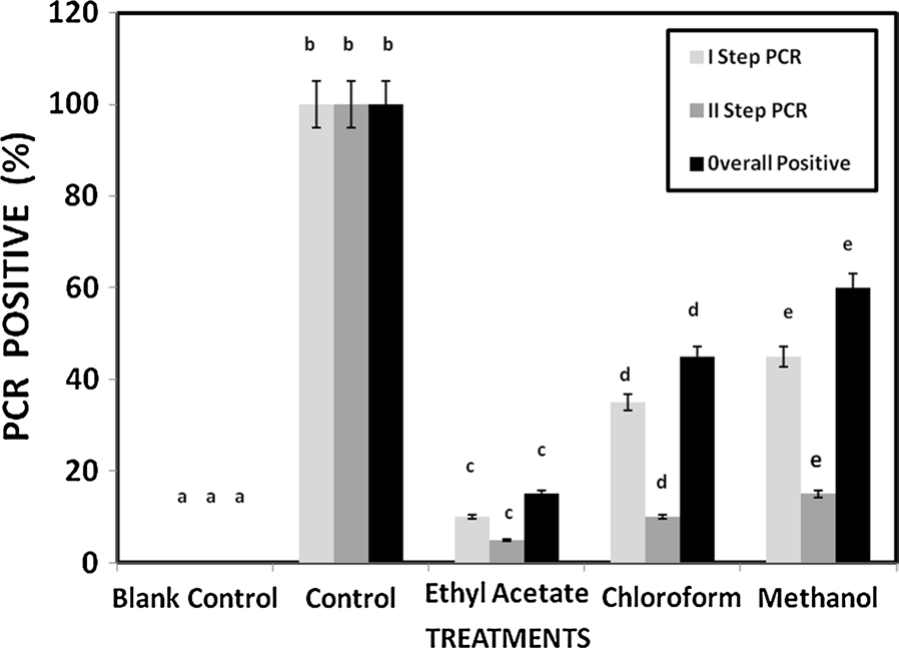
Antiviral activity performed by *F. indicus* injected with WSSV incubated the secondary metabolites of *Streptomyces* spp AJ8. Antiviral activity determined by double step diagnostic PCR detection. Means with the same superscripts (**a**–**e**) do not differ from each other (P < 0.001): one way ANOVA

**Fig. 6:**
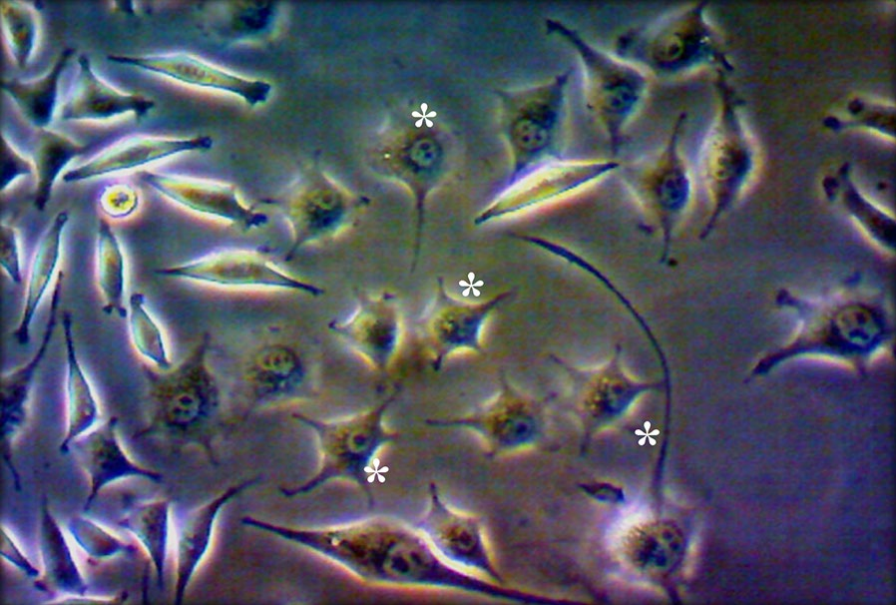
In vitro anticancer activity performed by MTT cell viability assay in L929 cell lines by the applying *Streptomyces* spp AJ8 secondary metabolites extracted from ethyl acetate. *Asterisk* indicates malformed cells

Among the different extractions the secondary metabolites which extracted from ethyl acetate were effectively suppressed the pathogenic bacteria of 19.2, 12.4, 10 mm of zone of inhibition in *S. aureus, A. hydrophila* WPD1 and *E. coli* respectively. The same metabolites also effectively suppressed the fungal growth the ranged between 8.5 and 10.3 mm of zone of inhibition against *A. niger, C. albicans* and *Pythium* spp. (Table 6). For antiviral activity, 100 % PCR positive signals were observed in the *F. indicus* when no secondary metabolites incubated WSSV injection given whereas the PCR signal was significantly (P < 0.001) reduced the secondary metabolites incubated WSSV injected shrimps. Among the secondary metabolites extracted from different solvents, ethyl acetate was effectively reduced the WSSV load by reflecting the week PCR signals after double step detection of only 15 %. The extraction also helps to reduce the WSSV load of 85 % from the control group (Fig. 5). Anticancer activity was performed in L929 fibroblast by treating with the secondary metabolites revealed that, the malformed cells seen in the cell culture after 24 h. After different concentration treated with the cancer cells, the secondary metabolites concentrations kill the L929 fibroblast cells at the rate of 75.23, 69.8 and 35.7 % in 100, 500 and 1000 µg/ml respectively and significantly (P < 0.001) differed (Table 7; Fig. 6).

**Table 6:** Antimicrobial screening of *Streptomyces* spp AJ8 secondary metabolites against bacterial and fungal pathogens

Microbial pathogens	Zone of inhibition (mm)		
	Ethyl acetate	Chloroform	Methanol
*Escherichia coli*	10.0 ± 0.01	7.8 ± 0.01	6.1 ± 0.01
*Staphylococcus aureus*	19.2 ± 0.1	6.4 ± 0.02	2.4 ± 0.4
*Pseudomonas aeruginosa*	6.0 ± 0.2	4.1 ± 0.3	5.4 ± 0.01
*Aeromonas hydrophila* WPD1	12.4 ± 0.1	2.4 ± 0.04	5.09 ± 0.05
*Vibrio harveyi*	8.2 ± 0.05	4.09 ± 0.02	3.4 ± 0.4
*Vibrio parahaemolyticus*	5.06 ± 0.04	3.2 ± 0.05	2.7 ± 0.03
*Aspergillus niger*	8.5 ± 0.5	2.5 ± 0.5	5.2 ± 10
*Candida albicans*	10.3 ± 0.02	–	–
*Pythium* spp.	7.4 ± 0.9	–	–

**Table 7:** In vitro anticancer activity performed by MTT cell viability assay in L 929 cell lines by the applying *Streptomyces* spp AJ8 secondary metabolites

Sl. no	Concentration (µg/ml)	OD (540 nm)	% viability
1	Control	0.630	100^a^
2	100	0.474	75.23^b^
3	500	0.440	69.8^c^
4	1000	0.225	35.7^d^

^a–d^Means with the same superscript do not differ from each other (P < 0.001): one way ANOVA

## Discussion

Haloalkaliphilic actinomycetes are a kind of extreme environment actinomycetes, which are highly tolerant to high NaCl concentration, as well as high pH which yielding to valuable industrial products including antimicrobials (Cai et al. 2009). Due to higher stability and tolerance of *Streptomyces* spp. AJ8 in extreme salinity and pH, their antimicrobial products are very effective against tested pathogens and they inhibit more than 10 mm of zone of inhibition. *Streptomyces* spp SBU1 isolated from the saltpan regions of Cuddalore, Tamilnadu, India showed most promising antagonistic activity against *E. coli*, *Pseudomonas aeruginosa* and *S. aureus* (Sudhakar et al. 2012). Moreover Aruljose et al. (2013) isolated and proved the antagonistic activity of polyketide producing halophilic *Streptomyces* spp. JAJ06 from the Tuticorin saltpan soils, India.

Halophilic actinomycetes including *Streptomyces, Saccharopolyspora, Micromonospora, Nocardia, Nocardiopsis,* and *Nonomuraea* were isolated identified from saltern ponds of Tuticorin, India (Aruljose and Jebakumar 2012). Interestingly some of the halophilic actinomycetes strains had showing optimum growth at 15 % NaCl was also found in salt lakes of Xinjiang, China (Cao et al. 2009). The present study the *Streptomyces* spp. AJ8 was well grown in the Knight’s agar media containing 10 % NaCl concentration. They also ferment glucose, sucrose, mannitol, starch and sorbitol etc. Two new species of *Streptomonospora,* named as *Streptomonospora amylolytica* and *S. flavalba* were isolated on starch-casein agar medium having 10 % NaCl (Cai et al. 2009). Our earlier study *Streptomyces* spp. AJ7, AJ9 and AJ10 which isolated from solar salt works were well grown in Starch casein agar, Tryptone yeast extract agar (ISP7) and Knight’s agar and produced the mycelium of light ash to white colour. They were also tolerated the pH of maximum 8.5 and grown well in 4 % NaCl (Jenifer et al. 2013).

The 16S rRNA sequencing has been widely used as a molecular clock to estimate relationships among the microbes, but more recently it has also become important as a means to identify unknown microbes up to the species level (Sacchi et al. 2002). In our study, 16S rRNA sequencing tools helped to identify the *Streptomyces* spp AJ8 and it was more than 90 % identical to other *Streptomyces* spp. including *Streptomyces* spp. 6G16, *Streptomyces* spp. SCAUKO356, *Streptomyces* spp 337702 and *S. fragilis* YJ-RT6. Sadeghi et al. (2014) isolated the salt tolerant *Streptomyces* spp. C-2012 from the Iranian soil, identified by taxonomic level using 16S rRNA gene sequencing and it revealed that, closely related to *S. rimosus* JCM 4667T. The sequence data for the 16S rRNA gene is highly conserved for different organisms and has also been shown to be very accurate for genus and species identification of bacteria and actinomycetes. Actinomycetes represent one of the most important sources for the discovery of new metabolites with biological activity; and many of these are described as being produced by polyketide synthases (PKS) and NRPS (Ayuso et al. 2005). In our study, the presence of NRPS gene cluster at the size of around 721 bp in the *Streptomyces* spp. AJ8, it may have potent antimicrobial and antitumor activities. The gene cluster involved the different pharmacological activities including antimicrobial, anti cancer and other activities proved by many authors. Mo et al. (2011) cloned and characterized a biosynthetic gene cluster, tirandamycin from marine-derived *Streptomyces* spp. SCSIO1666 with potential bacterial RNA polymerase inhibitory activity. Virginiamycin M (VM), a hybrid polyketide-peptide antibiotic had potent antibacterial activity was characterized from *S. virginiae* (Pulsawat et al. 2007). Arasu et al. (2012) also identified a polyketide metabolite had the antibacterial, antifungal and anticancer activities isolated from the marine *Streptomyces* spp. AP-123 isolated from Andra Pradesh, India. Moreover, the acetylcholinesterase (AChE) enzyme binding site was observed in the NRPS gene. The AChE belongs to carboxylesterase family of enzymes that hydrolyzes the neurotransmitter acetylcholine. The AChE inhibitors were characterized from the compounds including arisugacins A and B which obtained from Penicillium sp. FO-4259 (Otogwo et al. 1997).

Our previous study by Jenifer et al. (2013) described that, *Nocardiopsis* spp. AJ1 and *Streptomyces* spp. AJ7 isolated from solar salt works had effectively controlled various aquatic and human pathogenic bacteria. Extremophilic actinomycetes are also considered as an unexplored source of antifungal compounds (Wu and Zhang 2007). In our studies, the ethyl acetate extracts were effectively suppressed the bacterial and fungal pathogens due to the antimicrobial compounds present in the metabolites. Due to the mid polarity of the ethyl acetate extraction, most of the polar and mid polar compounds active compounds eluted these solvents. A moderately halophilic *Streptomyces* spp. JAJ06 producing an antimicrobial compound of polyketide type was isolated from saltpan soil collected at Tuticorin, India (Aruljose et al. 2011). Antifungal secondary metabolites have been isolated from alkaliphilic *N. dassonvillei* WA52 (Ali et al. 2009), Nocardia spp. ALAA 2000 (El-Gendy et al. 2008) and marine Streptomyces spp. DPTB16 (Dhanasekaran et al. 2008). *S. violaceusniger* G10 produced antifungal metabolites that effectively inhibited the growth of Fusarium spp. (Getha and Vikineswary 2002). Antiviral activity of halotolerant actinomycetes is also reported against tobacco mosaic tobamovirus and potato Y potyvirus (Mohamed and Galal 2005). In our studies, ethyl acetate was effectively reduced the WSSV load by reflecting the week PCR signals after double step detection of only 15 %. The secondary metabolites of *Streptomyces* spp. AJ8 was inhibit the transcription and translation of the WSSV leading to arrest the viral multiplication. Serkedjieva et al. (2012) characterized a novel proteinaceous protease inhibitor from *S. chromofuscus* 34-1 (SS34-1) demonstrated a specific and selective anti-influenza virus effect. Furan-2-yl acetate, an antiviral compound extracted from marine *Streptomyces* sp VITSDK1 was effectively controlled the fish nodavirus (FNV) (Suthindhiran et al. 2009). The secondary metabolites concentrations kill the L929 fibroblast cells up to 35 % maximum in highest concentrations. The secondary metabolites from *Streptomyces* spp RJ8 expressed their highest anticancer activity (65 %) at 1000 µg/ml concentration against L 929 Fibroblast cell lines (Remya 2013). Pyrocoll, an antitumour compound was recently detected in novel alkaliphilic *Streptomyces* strain (Dietera et al. 2003). The moderately halophile *Saccharopolyspora salina* VITSDK4 produces an extracellular compound with cytotoxicity on HeLa cells that show the IC50 value of 26.2 µg/ml (Du et al. 2012). The secondary metabolites extracted from the *Streptomyces* spp. AJ8 had the potent antimicrobial and anticancer properties. The metabolites were highly influenced to control various pathogenic bacteria, fungi and the killer shrimp virus WSSV. The cluster gene NRPS was also successfully amplified from the genomic DNA of *Streptomyces* spp. AJ8 indicated that the NRPS gene responsible for the antimicrobial and anticancer activities. Further study need to sequencing the whole genome of the *Streptomyces* spp. AJ8 to find out the pharmacological important active compounds.
